# Valacyclovir in the treatment of acute retinal necrosis

**DOI:** 10.1186/1471-2415-12-48

**Published:** 2012-09-05

**Authors:** Simon RJ Taylor, Robin Hamilton, Claire Y Hooper, Lavnish Joshi, Jiten Morarji, Nitin Gupta, Sue L Lightman

**Affiliations:** 1 Division of Immunology & Inflammation, Faculty of Medicine, Imperial College London, London, UK; 2 Royal Surrey County Hospital NHS Foundation Trust, Guildford, UK; 3 Moorfields Eye Hospital, London, UK; 4 UCL Institute of Ophthalmology, London, UK

**Keywords:** Acute retinal necrosis, Herpetic retinitis, Acyclovir, Valacyclovir

## Abstract

**Background:**

To report the outcome of oral valacyclovir as the sole antiviral therapy for patients with acute retinal necrosis (ARN).

**Methods:**

This study reports a retrospective, interventional case series of nine consecutive patients with ten eyes with newly diagnosed ARN treated with oral valacyclovir as the sole antiviral agent. Eight patients received oral valacyclovir 2 g tid (Valtrex, GlaxoSmithKline) and one patient with impaired renal function received oral 1 g tid. The main outcome measures were response to treatment, time to initial response to treatment, time to complete resolution of retinitis, best corrected visual acuity (BCVA) at final follow-up, retinal detachment and development of recurrent or second eye disease.

**Results:**

Retinitis resolved in ten of ten (100%) affected eyes. The median time to initial detectable response was seven days and the median time to complete resolution was 21 days. A final BCVA of 20/40 or better was achieved in 6/10 (60%) of eyes. 3/10 eyes (30%) developed a retinal detachment. No patients developed either disease reactivation or second eye involvement over the course of the study (mean follow up 31 weeks, range 7 to 104 weeks).

**Conclusions:**

Treatment with oral valacyclovir as the sole antiviral therapy resulted in complete resolution of retinitis. Final BCVA and retinal detachment rate were comparable with previously reported outcomes for intravenous acyclovir.

## Background

Acute retinal necrosis (ARN) is a rare, but potentially devastating, syndrome characterised by progressive peripheral necrotising retinitis. It was first described in 1971 [1], but it took more than a decade later for its herpetic aetiology to be discovered and antiviral therapy to become the mainstay of treatment [2,3]. The current standard of care for ARN consists of intravenous acyclovir 10 mg/kg (or 1500 mg/m^2^) every 8 h for 5–10 days, followed by oral acyclovir 400-800 mg 5 times daily for an additional 6-12 weeks, to reduce the risk of second eye involvement [4]. Nevertheless, there is a lack of consensus concerning the treatment of ARN, with an increasing number of groups reporting primary treatment of ARN with oral antiviral therapy alone [5-10], although others suggest that intravenous antiviral therapy is superior [11] or even that higher doses of intravenous therapy are indicated in most patients without renal impairment [12].

Advocates of oral antiviral therapy argue that the adoption of intravenous antiviral therapy predated the discovery of valacyclovir and famciclovir, both of which have superior plasma bioavailability to acyclovir as oral preparations [13]. Both agents have been reported to be successful in the treatment of ARN in small case series [5-8,14]. This study adds to this evidence and is, to our knowledge, the largest consecutive case series of patients with ARN treated solely with oral valacyclovir without antecedent intravenous therapy or adjunctive intravitreal therapy.

## Methods

The study consisted of a retrospective review of the medical records of nine consecutive patients who presented between December 2006 and November 2009 at a single tertiary referral centre with a new diagnosis of ARN and whose antiviral therapy consisted solely of oral valacyclovir. This study was approved by the Research Governance Committee of Moorfields Eye Hospital (LIGS1023: Visual loss in uveitis). The clinical diagnosis of ARN was based on the diagnostic criteria established by the American Uveitis Society, which consist of: (1) one or more discrete foci of peripheral retinal necrosis; (2) occlusive retinal vasculitis; (3) acute panuveitis; (4) circumferential disease spread; (5) rapid progression of disease in the absence of treatment [15]. All patients had active, not indolent disease, as defined by the presence of significant uveitis. In three out of nine cases, the clinical diagnosis was confirmed by demonstration of herpetic viral DNA in aqueous or vitreous samples by polymerase chain reaction (PCR); in the remaining six cases, the diagnosis of presumed herpetic retinitis was made on the basis of the above clinical diagnostic criteria alone.

The extent of retinal involvement was recorded using the same classification scheme as for cytomegalovirus retinitis [16]. In this scheme, zone 1 comprises a circle with a 3000 μm radius centred on the fovea, with an extension 1500 μm nasally from the optic nerve head, zone 2 extends from the anterior border of zone one to the equator, and zone 3 extends anteriorly from the equator to the ora serrata.

All patients were treated as outpatients. Patients received oral valacyclovir 2 g tid (Valtrex, GlaxoSmithKline), unless there was evidence of renal impairment, in which case the dose was reduced to 1 g tid, and were treated for a minimum duration of 6 weeks. Where there was significant vitritis or optic nerve involvement, such that visual acuity was impaired, systemic corticosteroid therapy at a dose of 0.5-1 mg/kg was instituted on the same day as antiviral therapy, and was tapered over the course of the antiviral therapy. Renal function was monitored closely throughout treatment. Prophylactic laser treatment consisted of three consecutive rows of confluent and circumferential laser applied to normal retina posterior to the edge of the necrotic retina, and was performed if there was adequate visualisation of the fundus and sufficient uninvolved retina to allow application of laser without endangering the optic nerve or macula.

Outcome measures included time to clinical response, time to complete resolution of retinitis and best corrected visual acuity (BCVA) at the final follow-up visit. Clinical response was defined as the fading of areas of retinitis. Complete resolution was defined as the total disappearance of active retinitis and vasculitis, together with evidence of retinal pigment epithelial atrophy or pigmentation. The development of retinal detachment, and the occurrence of second eye involvement or disease reactivation were also assessed.

## Results

The study included ten eyes of nine patients; one patient with HIV co-infection which was being successfully treated with HAART (CD4 count > 500 cells/mm^3^ and undetectable viral load) presented with disseminated herpes zoster virus infection and bilateral ARN. Patient and disease characteristics and the results of treatment are summarised in Table 1. The median age at disease onset was 38 years (range 30 to 82 years) and the mean duration of symptoms prior to presentation was 14 days (range 1 to 28 days). Two patients were HIV positive (patients 6 and 8) but the remaining seven patients did not have a history suggestive of underlying immune dysfunction. All ten eyes had at least one discrete focus of peripheral retinal necrosis. Four eyes had zone two disease and three eyes had zone one disease. Three eyes had prominent vitritis and two of these eyes also had optic disc oedema.

**Table 1:** Clinical findings and response to treatment with oral valacyclovir

**Patient**	**Age,Sex**	**Eye**	**Virus**	**Posterior extent (zone)**	**Time to initial response (weeks)**	**Time to complete resolution (weeks)**	**Initial BCVA**	**Final BCVA**	**Laser**	**Steroids**	**Complications**
1	79, M	R	Undetermined	1	1	5	20/200	20/40	No – Zone 1	OFSI	RPE change at macula
2	39, M	L	Presumed VZV	3	1	1	20/20	20/20	No – Zone 1	No	Nil
3	82, M	R	VZV on PCR	1	1	4	20/120	CFs	Yes	Oral prednisolone	RD at 45 days
4	54, F	L	VZV on PCR	2	2	6	CFs	20/60	Yes	Oral prednisolone	Nil
5	36, M	R	HSV-2 on PCR	2	1	3	20/30	20/80	Yes	Oral prednisolone	RD at 35 days
6	36, M	L	Undetermined	3	1	3	20/20	20/16	No	No	Nil
7	33, M	L	Presumed HSV-1	1	2	5	HMs	LP	No – Zone 1	Oral prednisolone	RD at 49 days
8	30, F	R	Presumed VZV	3	1	2	20/20	20/20	Yes	No	Nil
		L	Presumed VZV	2	1	2	20/30	20/20	Yes	No	Nil
9	81, M	L	Undetermined	2	1	6	20/20	20/40	Yes	Oral prednisolone	Nil

*VZV* = Varicella zoster virus; *HSV* = Herpes simplex virus; *PCR* = polymerase chain reaction; *BCVA* = best corrected visual acuity; *CFs* = count fingers; *HMs* = hand movements; *LP* = perception of light; *OFSI* = Orbital floor corticosteroid injection; *RD* = retinal detachment.

Varicella zoster virus (VZV) was identified as the most likely causative virus in five eyes of four patients. Two cases were proven on PCR, and it was the presumed cause in two further patients, one of whom had disseminated herpes zoster at the time of presentation (patient 8) and one of whom had a history of recent chicken pox infection (patient 2). One patient had HSV-2 infection proven by PCR, and one patient was presumed to have HSV-1 infection owing to a past history of HSV-1 encephalitis.

All patients were treated with oral valacyclovir for at least six weeks. One patient with HIV infection and disseminated VZV was advised to remain on maintenance therapy, but was lost to follow up after seven weeks. Maintenance therapy was discussed with the patient who had had HSV encephalitis as an infant, but this was declined.

**Figure 1:**
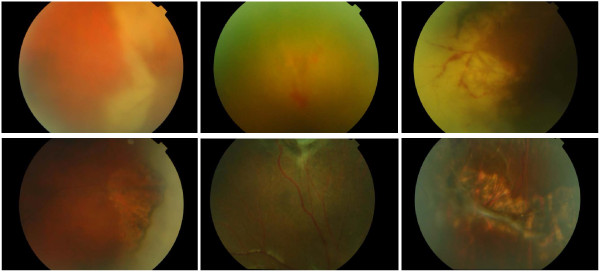
**Representative images from three patients with acute retinal necrosis treated with oral valacyclovir 2 g tds as their sole antiviral therapy.** Images from the top row are pre-treatment and correspond to post-treatment images in the bottom row.

The median time to initial response was 7 days (range 7-14 days) and retinitis completely resolved in all ten eyes (100%) in a median 21 days (range 7-42 days). Representative images from three patients are included in Figure 1. Four patients received adjunctive systemic corticosteroids, and one patient received a single orbital floor injection of 40 mg triamcinolone as an alternative to systemic corticosteroid therapy at presentation. Six eyes were treated with prophylactic barrier laser; this was not possible in three eyes as they had zone one disease and, in one case, laser was considered unnecessary owing to the area of retinitis being small.

6/10 eyes (60%) achieved a final BCVA of 20/40 or better, but 2/10 eyes (20%) were 20/200 or worse by final follow-up. Three eyes developed retinal detachments at 35-49 days following initiation of treatment. Two patients underwent pars plana vitrectomy, endolaser and silicone oil, and one patient declined surgical intervention. There was no evidence of disease reactivation or second-eye involvement over the follow-up period of a median 31 weeks (range 7-104 weeks).

## Discussion

In this series, we report the results of treatment of ARN with oral valacyclovir, and believe this to be the largest such consecutive series published to date. Valacyclovir proved effective at treating retinitis in all ten eyes, and the median times to initial response and complete resolution of retinitis were 7 days and 21 days respectively, which is similar to the results of other series in which intravenous acyclovir [2,17] or other oral antiviral agents [5] were used. Similarly, 60% of our patients achieved a final BCVA of 20/40 or better and 20% had a final BCVA of 20/200 or worse; both comparable with previous reported series of oral antiviral agents [5,17] and intravenous acyclovir [18-20].

We recorded no cases of fellow eye involvement. The natural history of ARN is for some two thirds of fellow eyes to become involved, although intravenous acyclovir has reduced this to 3-14% [14,18,19,21-23]. Other series in which oral antiviral agents were used have also failed to report fellow eye involvement [5], but involvement can occur decades later [24,25], so longer follow up would be required to confirm this finding. 30% of our patients developed a retinal detachment. The natural history of ARN is for 75% of affected eyes to progress to retinal detachment [26], the reported rate with intravenous acyclovir being reduced to 20-52% [14,17-20,22]. Most retinal detachments occur within six months of presentation [14], and 6 of 8 patients in our series had follow-up longer than six months, suggesting that the rate is unlikely to be significantly underreported.

There is a wide variation in the reported 50% inhibitory concentrations (IC_50_) of acyclovir *in vitro* – 0.02 to 13.5 μM for HSV-1, 0.01 to 9.9 μM for HSV-2, and 0.12 to 10.8 μM for VZV [27-29]. Administration of oral valacyclovir 1 g tid has been shown to achieve plasma concentrations in excess of the IC_50_ for most clinical isolates of HSV-1, HSV-2 and VZV throughout the dosage period [28], as has administration of famciclovir 500 mg tds [30], but this dose still only achieves daily acyclovir area under the concentration-time (AUC) values similar to those obtained with intravenous acyclovir 5 mg/kg tid [31,32]. In contrast, valacyclovir 2 g tid produces a daily AUC of 109 μg·h^-1^ mL^-1^ which is similar to that achieved by intravenous acyclovir 10 mg/kg tid at 107 μg·h^-1^ mL^-1^[33].

We treated our patients with oral valacyclovir 2 g tid on the basis that this would achieve AUC values similar to intravenous acyclovir 10 mg/kg tid without exposing patients to the side-effects associated with higher doses of valacyclovir, which include the development of thrombotic microangiopathy [34], and without compromising patient compliance throughout a long treatment duration [35]. The 2 g tid dose was well tolerated in our patients, and no patient developed systemic adverse effects associated with treatment [32,35].

Limitations of this study include its retrospective nature and small sample size, which mean that it is not possible to comment on the efficacy or otherwise of oral prednisolone or prophylactic barrier laser. The short duration of follow up may also skew incidence rates for reactivation of disease, fellow eye involvement and retinal detachment. Nevertheless, treatment with oral valacyclovir 2 g tid appears to be as efficacious as previously reported outcomes with other antiviral treatments, and suggests that this is a valid treatment option for patients with acute retinal necrosis.

## Conclusions

Treatment with oral valacyclovir 2 g tid appears to be as efficacious as previously reported outcomes with other antiviral treatments, and suggests that this is a valid treatment option for patients with acute retinal necrosis.

## Competing interest

SRJT was supported by the UK National Institute of Health Research and by the Imperial NIHR Comprehensive Biomedical Research Centre. The authors report no conflicts of interest.

## Authors’ contribution

ST and SL designed the study. RH, CY, ST, LJ and NG collected data. ST, LJ and JM performed data analysis. RH, CY and NG drafted the manuscript. ST, LJ, JM and SL revised the manuscript. All authors read and approved the final manuscript.

## Summary

This study reports the outcome of oral valacyclovir as the sole antiviral therapy in ten eyes of nine patients with acute retinal necrosis. Retinitis resolved in all affected eyes and 60% of eyes achieved 20/40 or better. The retinal detachment rate was 30%, but no patients developed disease reactivation or second eye involvement.

## Pre-publication history

The pre-publication history for this paper can be accessed here:

http://www.biomedcentral.com/1471-2415/12/48/prepub
